# Electroacupuncture pretreatment ameliorates anesthesia and surgery-induced cognitive dysfunction in aged rats: insights from gut microbiota modulation

**DOI:** 10.3389/fmicb.2025.1642337

**Published:** 2025-12-01

**Authors:** Huajuan Lei, Ziou Li, Juan Deng, Heng Lei, Honghui Li, Zhendong Ding

**Affiliations:** 1 Department of Anesthesiology, The First Hospital of Traditional Chinese Medicine of Hunan University, Changsha, China; 2 Graduate School, Hunan University of Chinese Medicine, Changsha, China; 3 Xiangtan Medical and Health Vocational and Technical College, Xiangtan, China; 4 Department of Orthopedic, The First Hospital of Traditional Chinese Medicine of Hunan University, Changsha, China; 5 Department of Anesthesiology, The Third Xiangya Hospital of Central South University, Changsha, China; 6 Postdoctoral Station of Pharmacology, The Third Xiangya Hospital, Central South University, Changsha, China

**Keywords:** electroacupuncture, propofol anesthesia, aged rats, postoperative cognitive dysfunction, gut microbiota

## Abstract

**Objective:**

This study aimed to investigate the effects of electroacupuncture (EA) on postoperative cognitive dysfunction (POCD) and gut microbiota in aged rats anesthetized with propofol.

**Methods:**

Forty 18-months-old male SD rats were randomly divided into four groups: Sham, Model (MD), Sham Electroacupuncture (JE), and Electroacupuncture (EA), with 10 rats in each group. The Sham group underwent a skin incision without surgery, while the MD, JE, and EA groups received propofol anesthesia followed by right tibial surgery. The EA group received electroacupuncture at Baihui, Shenmen, and bilateral Zusanli points for 5 days prior to surgery, while the JE group received acupuncture without electrical stimulation. Behavioral tests, including the Morris water maze and open field tests, were conducted at 1-, 2-, and 3-weeks post-surgery to assess cognitive function. Gut microbiota composition was analyzed using second-generation sequencing.

**Results:**

At 1-week post-surgery, the MD, JE, and EA groups showed longer latencies and fewer crossings in the behavioral tests. However, at 3- and 4-weeks post-surgery, the EA group exhibited significantly reduced latency and increased crossing times compared to the MD and JE groups. Gut microbiome analysis revealed that the EA group had a higher relative abundance of Bacteroidetes and Proteobacteria, and a reduced relative abundance of Unclassified Lactobacillaceae compared to the MD and JE groups.

**Conclusion:**

Electroacupuncture was associated with improved postoperative cognitive function in aged rats after propofol anesthesia and tibial surgery. These effects were accompanied by alterations in gut microbiota composition, suggesting a possible link with the gut–brain axis, although causality remains to be established.

## 1 Introduction

Postoperative Cognitive Dysfunction (POCD) is a common and serious complication following surgery (Deiner et al., 2017; Ding et al., 2025; Lai et al., 2021). POCD predominantly affects elderly patients over the age of 65 and manifests through a variety of symptoms, primarily cognitive impairments such as decreased attention, orientation, calculation ability, spatial imagination, thinking, memory, etc. Studies have shown that the incidence of POCD in elderly patients after non-cardiac surgery ranges from 6.8% to 31% within 7 days, which is approximately twice as high as in younger patients (Evered et al., 2011). The prevalence of POCD is highest for 3 months after surgery, with approximately 13%–15% of elderly patients experiencing it (Zhao Q. et al., 2024). As age increases, the duration of POCD tends to extend, leading to a higher likelihood of losing basic social and daily living abilities, significantly detriment the quality of life postoperative (Deiner et al., 2021; Monk et al., 2008; Newman et al., 2001).

The pathogenesis of POCD remains unclear so far (He et al., 2024; Zhou et al., 2023). Evidence has revealed that it is associated neuroinflammation, mitochondrial dysfunction, oxidative stress, blood-brain barrier damage, neurotrophic support impairment, synaptic damage and other complicated mechanisms (Deng et al., 2024; Ding et al., 2025; Ji et al., 2024; Lu et al., 2024; Nemoto et al., 2022; Yang et al., 2022; Zhang et al., 2024; Zhou et al., 2023). More specifically, current research suggests that anesthesia and surgical stress may induce neuroinflammation and neurodegenerative changes in the aging brain, leading to a series of cognitive dysfunction syndromes (Bhushan et al., 2022; Fan et al., 2016; Luo et al., 2019; Netto et al., 2018).

Notably, Inflammation is not only a hallmark of aging but also one of the potential key factors driving the aging process (Campisi et al., 2019). Pro-inflammatory factors released during inflammation, such as cytokines, chemokines, and growth factors, can damage cellular structures and functions, thus can lead to cellular senescence and apoptosis. Additionally, the increased expression of both pro-inflammatory and anti- inflammatory cytokines following anesthesia and surgery, could cross blood brain barrier (BBB), exacerbates neuronal cell damage and even cell death (Gao et al., 2017).

The gut microbiota is a complex microbial system involved in various metabolic pathways, signal transduction, and the regulation of the immune-inflammatory axis (Cai et al., 2022; Collins et al., 2023). Through the gut microbiome-gut-brain axis, the gut microbiota plays a critical role in the central nervous system. Dysbiosis of the gut microbiota can activate microglial cells, leading to neuroinflammation. Pan et al. (2023) found that surgery causes significant changes in the gut microbiota composition of elderly mice, which may serve as the underlying mechanism for POCD and postoperative delirium (Jiang et al., 2019). However, there have been few studies exploring whether electroacupuncture (EA) can regulate the gut microbiota and the imbalance between pro-inflammatory and anti-inflammatory cytokines to alleviate POCD.

Currently, there are no specific or effective medications or treatment methods in Western medicine for POCD. In response to the challenges faced by POCD treatment, EA therapies show promising in filling the gap in POCD prevention and treatment. Acupuncture has a history of thousands of years in China, particularly for its significant effects on the nervous system (Zhao F. Y. et al., 2024). Early intervention with acupuncture produces prompt outcomes, and its long-term efficacy remains consistent (Hershman et al., 2022). However, the underlying mechanisms of acupuncture are intricate and not yet fully elucidated. Acupuncture, especially in the form of electroacupuncture, has gained attention for its broad and effective application in preventing and treating functional neurological disorders, due to its precise therapeutic effects, ease of operation, low cost, and minimal side effects. Recent studies have shown that acupuncture can improve cognitive function through the gut-brain axis, drawing increasing attention (Yin et al., 2025).

Therefore, this study uses propofol anesthesia and tibial surgery to establish an aged rat model of POCD, with EA and sham EA pre-treatment. It aims to explore the protective effects of EA on cognitive function in POCD rats, aiming to examine the specific effects and differences of EA in modulating the gut microbiota, providing a reference for the development and application of EA in anti-aging therapies.

## 2 Materials and methods

All animal experiments were conducted in accordance with the Guidelines for the Care and Use of Laboratory Animals of China. All experimenters held valid certificates for animal handling training. The animal experimental protocol was approved by the Animal Review Committee of the First Affiliated Hospital of Hunan University of Chinese Medicine (Ethics Approval Number: ZYFY20230620-02).

### 2.1 Experimental animals

Forty healthy male SD rats, 18 months old, weighing 550–650 g, were used in the study. They were kept at a temperature of 22 °C–26 °C with humidity maintained at 50%–70%. The animals were provided by Hunan Slaike Jingda Company [License No. SCXK (Xiang): 2020-0010]. All rats were housed in a room with a constant temperature and had free access to water and food. After a 1-week acclimatization period, the rats were randomly assigned to four groups using computer randomization (*n* = 10 per group): Sham group, Model group (MD), sham acupuncture group (JE), and Electroacupuncture group (EA). All animals were fed standard chow and marked with ear tags.

### 2.2 Main instruments and reagents

- Hua Tuo Electroacupuncture Device (GB2024-94, Model H)
- Hua Tuo Brand 1.0-inch Copper Acupuncture Needles (Shuzhou Medical Supplies Factory, Specifications: Diameter 0.35 mm × 50 mm)
- Morris Water Maze (Beijing Zhongshi Di Chuang Technology Development Co., Ltd., ZS-Morris, Number: 202100000000204)

### 2.3 Electroacupuncture intervention

**FIGURE 1:**
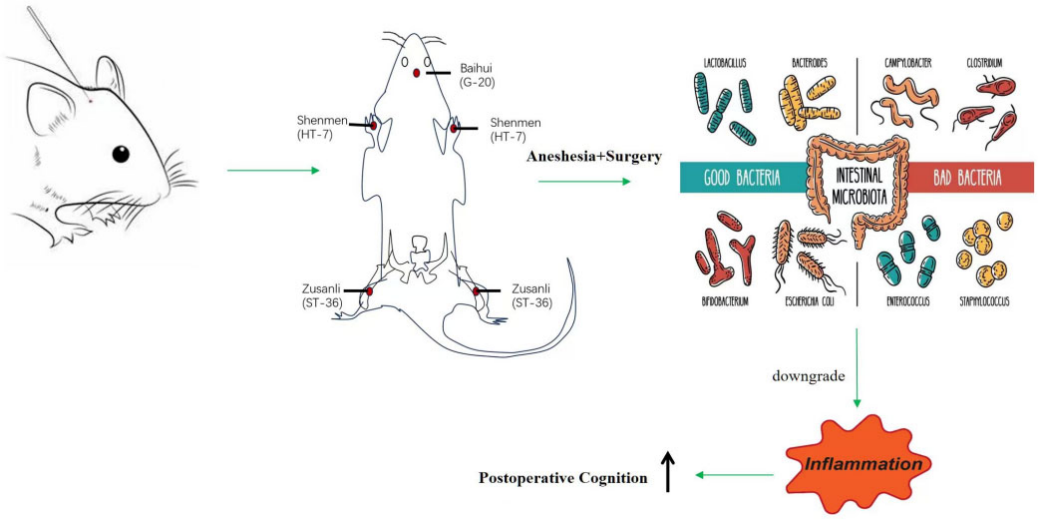
Diagram of electroacupuncture pretreatment ameliorates anesthesia and surgery induced cognitive dysfunction in aged rats.

**FIGURE 2:**
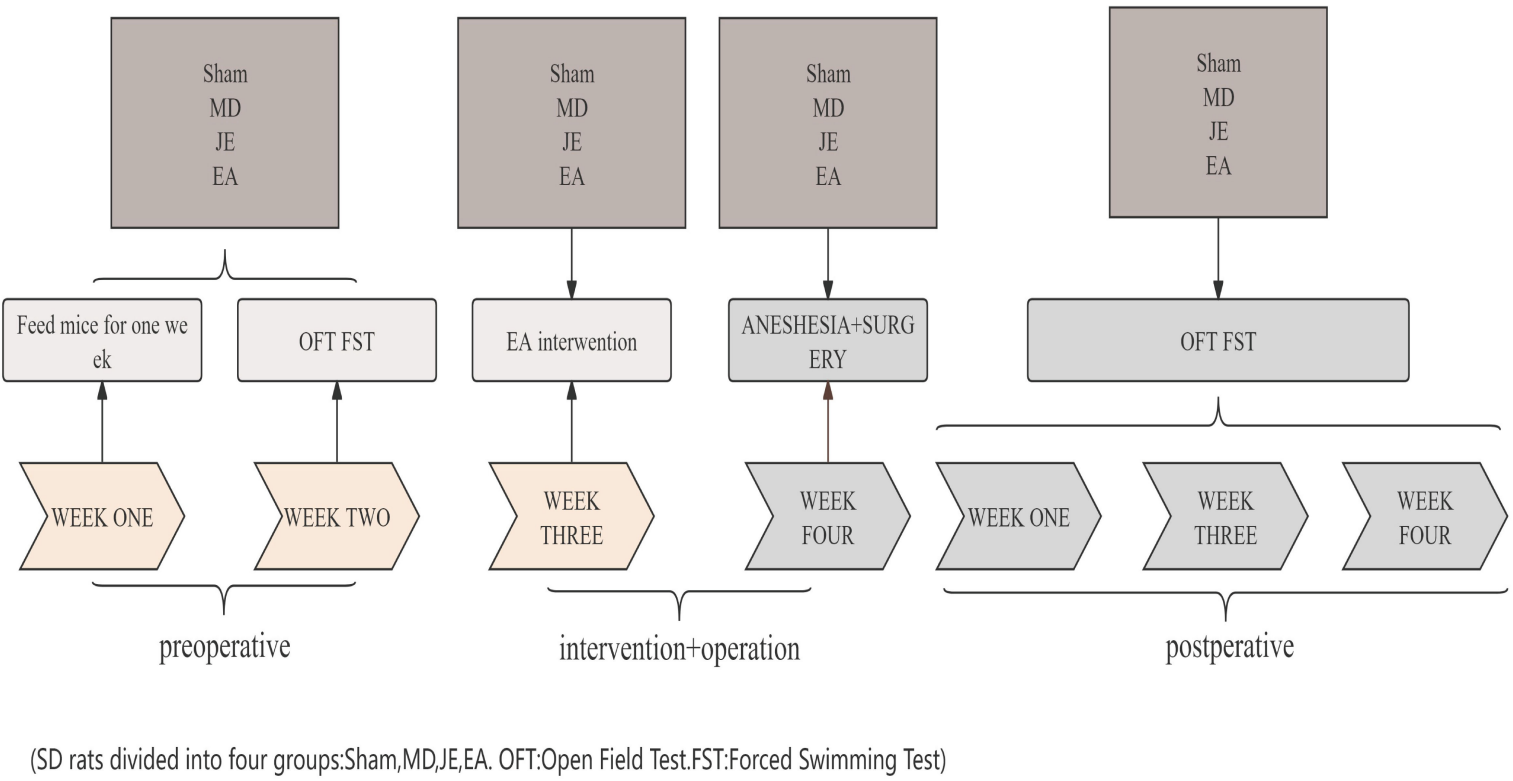
Timeline of electroacupuncture pretreatment ameliorates anesthesia and surgery induced cognitive dysfunction in aged rats flow chart.

A custom-made wooden fixation device with elastic straps was used to immobilize the rats’ limbs and head. The EA intervention protocol was as follows: The EA group started the treatment 5 days before surgery, using a Hua Tuo electroacupuncture device to stimulate the Baihui, Shenmen, and bilateral Zusanli acupuncture points (Figure 1). The acupuncture points were located according to Common Acupuncture Point Names and Locations in Laboratory Animals. The Baihui point is located at the midline of the parietal bone, the Shenmen point is on the inner side of the forelimb, at the ulnar margin of the wrist crease. A 30-gauge acupuncture needle connected to an electrode was inserted subcutaneously at a 45° angle to a depth of 2 mm. The Zusanli point is located on the outer side of the knee joint, between the tibia and fibula, about 3 mm from the head of the fibula. A 30-gauge needle connected to an electrode was inserted vertically to a depth of 7 mm. A slight tremor of the rat’s hind limb was considered an effective electroacupuncture response at the Zusanli point. Sparse-dense wave (5/15 Hz) with an intensity of 3 mA was applied for 30 min, once per day for 5 consecutive days. The JE group received acupuncture needles placed on the acupuncture points without electrical stimulation. The Sham and MD groups did not receive acupuncture or electrical stimulation. The overall experimental timeline is summarized in Figure 2.

### 2.4 Model establishment

24 h after the last EA pre-treatment, the rats in each group were weighed. The POCD model in aged rats was established using tibial fracture fixation. In the MD, JE and EA groups, the rats were anesthetized with propofol (Intraperitoneal injection, 30 mg/kg induction dose, followed by 15 mg/kg after 15 min) until the righting reflex disappeared and were placed in a supine position on a heated surgical table for fixation. Anesthesia was maintained with propofol, and the rats’ respiratory rate and the color of their noses and lips were continuously monitored. The right hindlimb was selected for surgery. The skin over the upper third of the tibia was shaved, and the area was disinfected with iodine. A sterile surgical drape was placed, and 2% lidocaine (7 mg/kg) was injected into the anterior lateral tibial shaft at the upper third. The skin was then incised about 1.5–2 cm in length, exposing the tibial shaft. A hole was drilled from the anterior edge of the tibial plateau to the tibial shaft, and a 5 mL syringe needle was inserted. Using an osteotomy scissor, the tibial shaft was cut at the upper third, resulting in a tibial fracture. The wound was cleaned and disinfected with iodine, and the skin was sutured. After the surgery, the rats were returned to individual cages, with attention to warmth and prevention of post-operative hypothermia. They were observed until they fully regained consciousness and were able to move. The Sham group underwent a similar procedure but only had the skin over the right hindlimb incised and sutured under propofol anesthesia without tibial fracture fixation. The surgical procedure was performed by the same person and took approximately 30 min.

### 2.5 Observation indicators and methods

#### 2.5.1 Morris water maze test (MWM)

The MWM system was used to assess the spatial learning and memory abilities of aged rats before tibial fracture surgery, and at 1 week, 3 weeks, and 4 weeks post-surgery. The average escape latency, target quadrant, and platform crossing times were analyzed. The water maze consists of a circular pool with a diameter of 120 cm, a depth of 50 cm, and a water depth of 30 cm, with a water temperature of (26 ± 2)°C. The pool is divided into four quadrants, and there are four entry points on the pool’s outer wall. A platform was placed in the fourth quadrant, submerged 1 cm below the water level and covered with black ink. The MWM experiment includes two main components: the navigation task and the spatial exploration task.

##### 2.5.1.1 Navigation task

The platform was placed in the center of the fourth quadrant, about 2 cm below the water surface. The rat was placed in the pool from any quadrant with its nose directed toward the pool wall. The time taken for the rat to find and climb onto the platform (escape latency) was recorded. If the rat could not find the platform within 90 s, it was guided to the platform and allowed to stay for 10 s. The escape latency was recorded as 90 s in this case. The rats were trained at fixed times, four times a day with a 30-s interval between each trial, for five consecutive days. On the 5th day, the swimming path and the time taken to find the platform (escape latency) were recorded as the results of the navigation task.

##### 2.5.1.2 Spatial exploration task

On the 6th day, the platform was removed. The rat was placed in the pool from any quadrant with its nose facing the pool wall. The swimming path, average swimming speed, and the number of times the rat crossed the platform’s previous location within 90 s were recorded as the results of the spatial exploration task.

#### 2.5.2 Open field test (OFT)

The OFT is used to evaluate an experimental animal’s autonomous behavior, exploratory activity, and anxiety levels in a novel environment. The open field test was conducted using an open observation box (100 cm × 100 cm × 40 cm), with the interior walls fully covered in black. The observation box was divided into 25 squares, with the central area occupying 9 squares in the middle and the surrounding squares designated as the W-border region. Before tibial fracture surgery, and at 1 week and 3 weeks post-surgery, the rats were gently placed at the center of the central area. Under bright lighting conditions, continuous tracking was performed using the open field system, and the total distance (in cm) of the rat within 5 min was recorded (Qi et al., 2018). A diagram of the open field test apparatus is illustrated in Supplementary Figure 1.

#### 2.5.3 Plasma IL-6, IL-1ββ, ROS enzyme-linked immunosorbent assay

Blood was collected into the procoagulant tube and the serum was prepared for standby. Serum concentrations of the inflammatory cytokines IL-6 and IL-1β, as well as the oxidative stress marker ROS, were determined using commercial ELISA kits (Jiangsu Jianglai Biotechnology Co., Ltd.).

#### 2.5.4 Gut microbiota 16S rRNA sequencing

After euthanizing the aged rats with 8% isoflurane anesthesia, the cecal contents were carefully collected using sterile forceps and placed into sterile sampling tubes for storage at −80 °C for later use. DNA was extracted from the samples for quality control and library preparation.

All samples were processed by Beijing Biomarker Biotechnology Co., Ltd., (Beijing, China). The total microbial genomic DNA was extracted from each sample following the instructions provided in the DNA extraction kit (MN NucleoSpin 96 So). The process included sample lysis, precipitation to remove impurities, filtering to eliminate inhibitors, DNA binding, washing, drying, and elution. The extracted total DNA was then purified using the Monarch DNA Gel Recovery Kit to recover PCR products. Finally, the PCR products were sequenced on the Illumina Novaseq 6000 sequencing platform.

### 2.6 Statistical methods

Statistical analyses were performed using SPSS version 26.0 (IBM, Armonk, NY, USA). Experimental data are expressed as mean ± standard deviation (’$\bar{\text{x}}$ ± s). Data distribution was first assessed for normality using the Shapiro–Wilk test, given the small sample size (*n* = 10 per group). For datasets meeting normality assumptions, one-way analysis of variance (ANOVA) was conducted. Homogeneity of variance was assessed with Levene’s test; if variances were equal, pairwise comparisons were performed using the least significant difference (LSD) test, while Dunnett’s T3 test was applied for unequal variances. For datasets violating normality assumptions, the Kruskal–Wallis test was employed as the non-parametric omnibus test, followed by Dunn’s *post hoc* procedure for pairwise comparisons with adjustment for multiple testing. A two-tailed *p*-value < 0.05 was considered statistically significant.

## 3 Results

### 3.1 Behavioral experiment results

#### 3.1.1 Escape latency task

**FIGURE 3:**
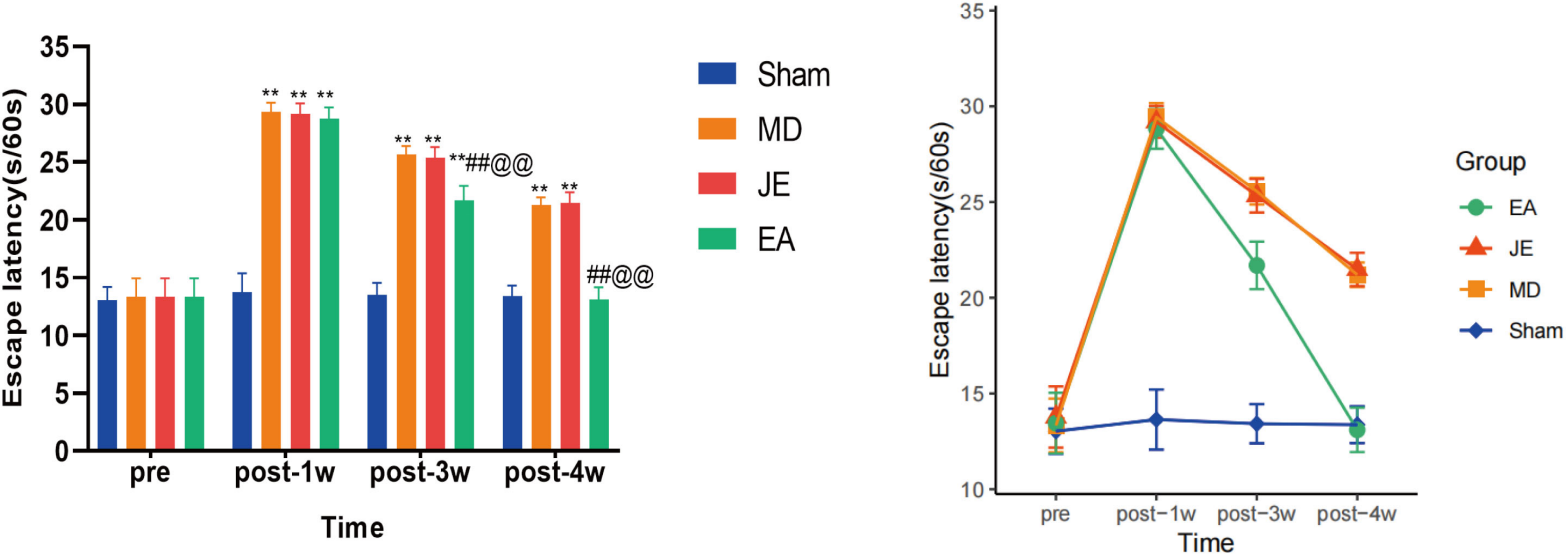
Histograms showing that escape latency of MWM in preoperative, 1 week, 3 weeks and 4 weeks postoperatively. The Sham group represents the sham surgery control group, the MD group represents the tibial fracture and propofol anesthsia model group, the JE group is the MD treated with sham electric-acupuncture, and the EA group is the MD pretreated with the electric-acupuncture (EA). The FST measures Escape Latency time, where longer periods of Escape Latency more severe cognitive decline behavior. (Compare with Sham group, **P < 0.01; compare with MD group, ^##^*P* < 0.01; compare with JE group,^@@^*P* < 0.01 *N* = 10/group). The experiment was repeated three times, and data were analyzed using repeated measures ANOVA.

As shown in Figure 3, at 1 week post-modeling, the escape latency in the MD and JE groups was significantly increased compared to the Sham group (*P* < 0.01), and the escape latency in the EA group was also significantly increased compared to the Sham group (*P* < 0.05), although the extent of increase was less severe than that in the MD and JE groups. Three weeks after modeling, the escape latency in the MD and JE groups decreased but was still significantly longer than that of the Sham group (*P* < 0.01). Four weeks after modeling, compared to the Sham group, the escape latency in the MD and JE groups remained significantly prolonged (*P* < 0.01). Furthermore, starting from 3 weeks post-surgery, the EA group exhibited a significantly shorter escape latency compared to the MD and JE groups (*P* < 0.01), and this effect persisted at 4 weeks (*P* < 0.01). Importantly, by the fourth week post-surgery, there was no longer a statistically significant difference in escape latency between the Sham and EA groups (*P* > 0.05).

#### 3.1.2 Spatial exploration task

**FIGURE 4:**
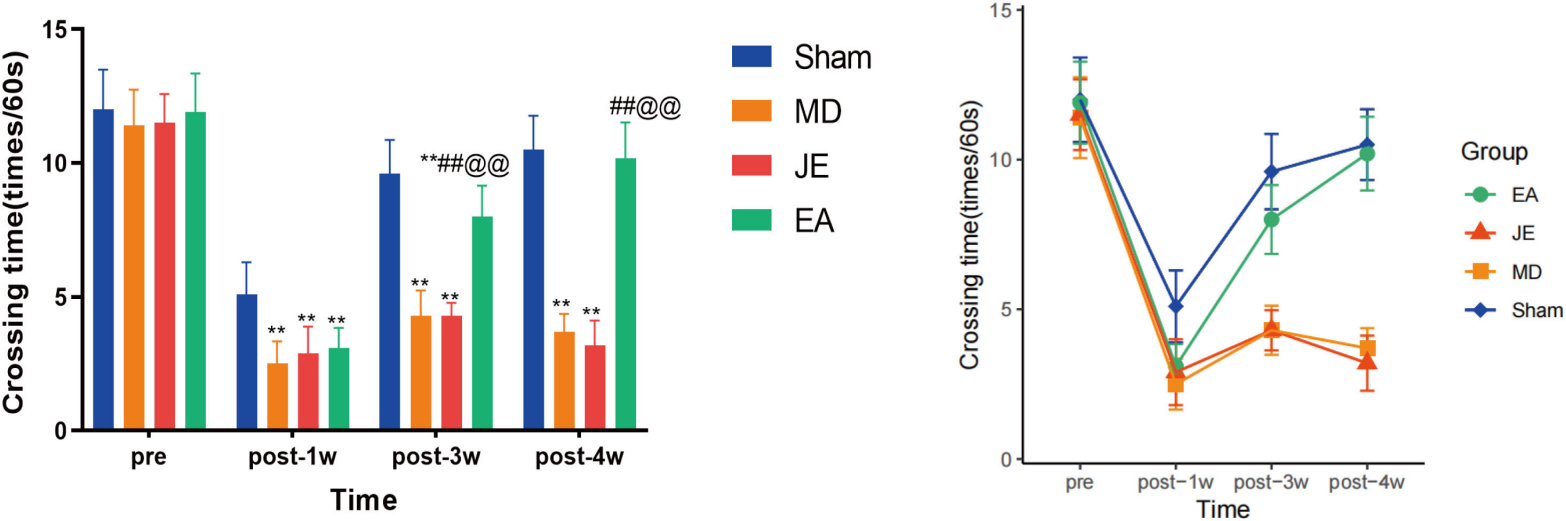
Histograms showing that crossing time of MWM in preoperative, 1 week, 3 weeks and 4 weeks postoperatively. The Sham group represents the sham surgery control group, the MD group represents the tibial fracture and propofol anesthsia model group, the JE group is the MD treated with sham electric-acupuncture, and the EA group is the MD pretreated with the electric-acupuncture (EA). The crossing time measures spatial exploration task, where the more times of spatial exploration task, the better spatial cognitive behavior. (Compare with Sham group, **P < 0.01; compare with MD group, ^##^*P* < 0.01; compare with JE group, ^@@^*P* < 0.01 *N* = 10/group). The experiment was repeated three times, and data were analyzed using Repeated Measures ANOVA.

The results of the number of platform crossings for each group (Figure 4) showed that, 1 week after modeling, the MD and JE groups had significantly fewer platform crossings compared to the Sham group (*P* < 0.01). From the third to the fourth week after modeling, the EA group, in contrast, showed a significant increase in platform crossings compared to both the MD and JE groups (*P* < 0.01). Three and four weeks after modeling, the number of platform crossings in all groups showed an increasing trend. Compared to the MD and JE groups, the Sham and EA groups had significantly more platform crossings (*P* < 0.01). The raw statistical results are available in Supplementary File 1.

### 3.2 OFT results for each group of rats

**FIGURE 5:**
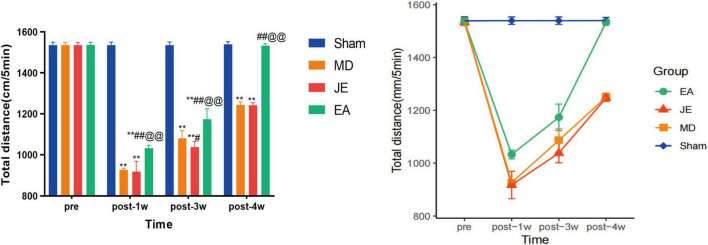
Histograms showing that total distance of OFT in preoperative, 1 week, 3 weeks and 4 weeks postoperatively. The Sham group represents the sham surgery control group, the MD group represents the tibial fracture and propofol anesthsia model group, the JE group is the MD treated with sham electric-acupuncture, and the EA group is the MD pretreated with the electric-acupuncture (EA). The total distance measures their locomotor activity and depressive-like behavior. A reduction in distance indicates depressive-like behavior, where the longer of total distance, the less depressive-like behavior. (Compare with Sham group, **P < 0.01; compare with MD group, ^#^*P* < 0.05, ^##^*P* < 0.01; compare with JE group, ^@@^*P* < 0.01 *N* = 10/group). The experiment was repeated three times, and data were analyzed using Repeated Measures ANOVA.

The results for total distance of OFT in each group (Figure 5) showed that, 1 week after modeling, the MD, JE, and EA groups all exhibited a decrease in total distance compared to the Sham group (*P* < 0.05), with the MD and JE groups showing a significant reduction. Compared to the MD and JE groups, the EA group showed a significant increase in total distance, with a statistically significant difference (*P* < 0.05). Three weeks after modeling, the total distance in the MD and JE groups was still shorter compared to the Sham and EA groups, with a statistically significant difference (*P* < 0.01) (Figure 5).

**FIGURE 6:**
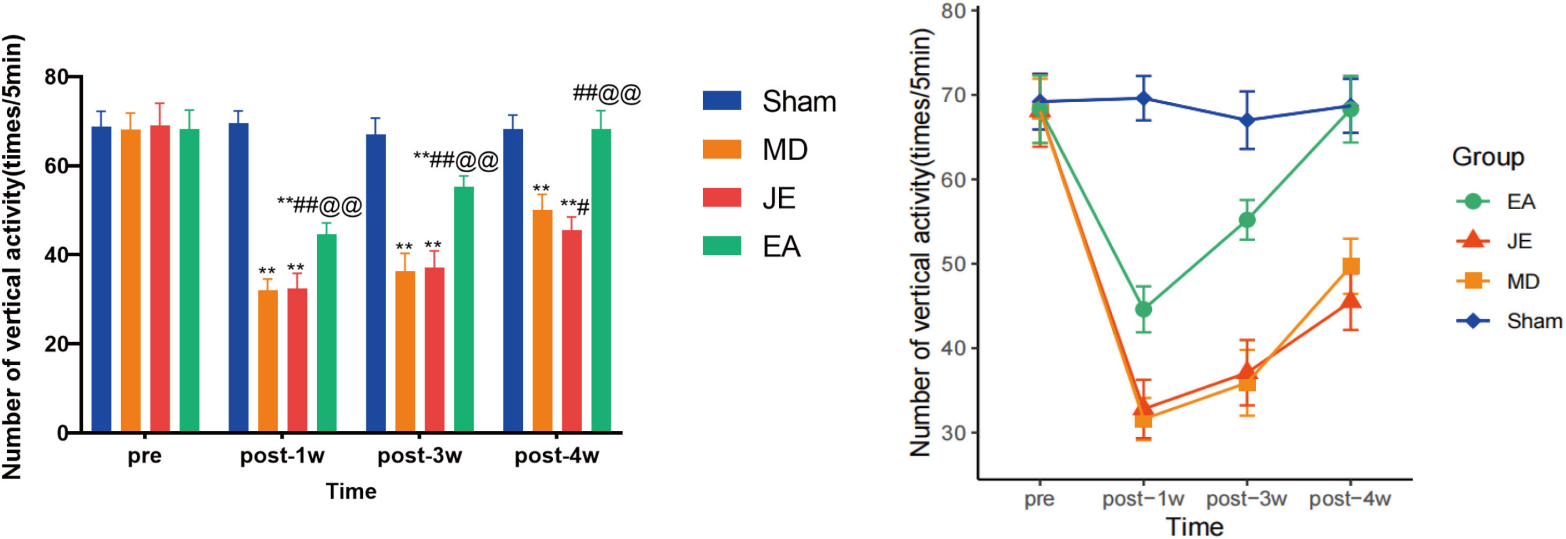
Histograms showing that total distance of OFT in preoperative, 1 week, 3 weeks and 4 weeks postoperatively. The Sham group represents the sham surgery control group, the MD group represents the tibial fracture and propofol anesthsia model group, the JE group is the MD treated with sham electric-acupuncture, and the EA group is the MD pretreated with the electric-acupuncture (EA). The total distance measures their locomotor activity and depressive-like behavior. A reduction in distance indicates depressive-like behavior, where the longer of total distance, the less depressive-like behavior. (Compare with Sham group, **P < 0.01; compare with MD group, ^#^*P* < 0.05, ^##^*P* < 0.01; compare with JE group, ^@@^*P* < 0.01 *N* = 10/group). The experiment was repeated three times, and data were analyzed using Repeated Measures ANOVA.

The results for numbers of vertical activity of OFT in each group (Figure 6) showed that, 1 week, 3 weeks after modeling, the MD, JE, and EA groups all exhibited a decrease in vertical activity compared to the Sham group (*P* < 0.01), 4 weeks after modeling, the numbers of vertical activity in the MD and JE groups was still less compared to the Sham, with a statistically significant difference (*P* < 0.01) and EA groups shows no difference compare with the Sham (*P* > 0.05). The raw statistical results are available in Supplementary File 2.

### 3.3 Comparison of right quadriceps circumference in each group of rats

**FIGURE 7:**
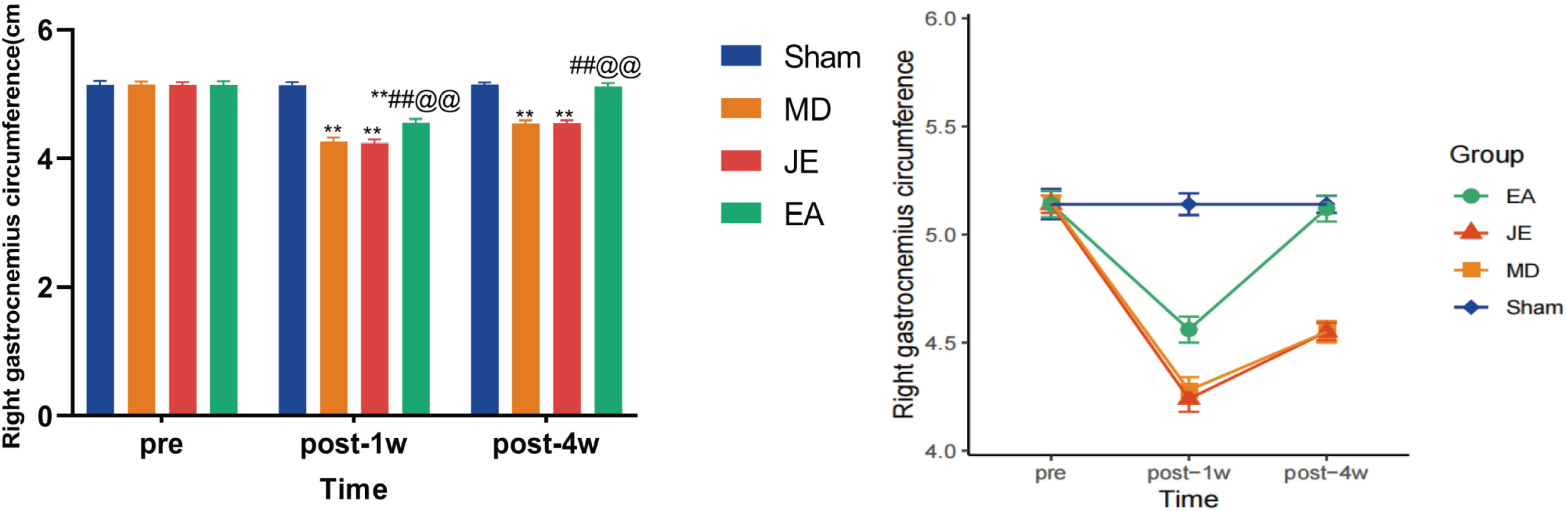
Histograms showing that right quadriceps circumference (RQC) in preoperative, 1 week and 4 weeks postoperatively. The Sham group represents the sham surgery control group, the MD group represents the tibial fracture and propofol anesthsia model group, the JE group is the MD treated with sham electric-acupuncture, and the EA group is the MD pretreated with the electric-acupuncture (EA). The Right Quadriceps Circumference measures their right tibial locomotor activity and muscle recovery. A reduction in RQC indicates weaker locomotor activity of the right tibia and less muscle recovery. (Compare with Sham group, **P < 0.01; compare with MD group, ^##^*P* < 0.01; compare with JE group, ^@@^*P* < 0.01 *N* = 10/group). The experiment was repeated three times, and data were analyzed using repeated measures ANOVA.

As shown in Figure 7, compared to the preoperative measurement of the right quadriceps circumference in rats, the circumference significantly decreased in the MD, JE, and EA groups at 1 week postoperatively, indicating notable muscle atrophy in the right leg (*P* < 0.01). However, after electroacupuncture preconditioning and 4 weeks of passive exercise rehabilitation through the MWM test, the EA group showed significant muscle recovery, with no statistically significant difference in thigh circumference compared to the Sham group (*P* > 0.05). In contrast, the MD and JE groups still exhibited smaller thigh circumferences than the Sham group, suggesting that muscle atrophy in the right leg had not fully recovered (*P* < 0.01). The raw statistical results are available in Supplementary File 3.

### 3.4 Comparison of inflammation marker in four groups of rats

**FIGURE 8:**
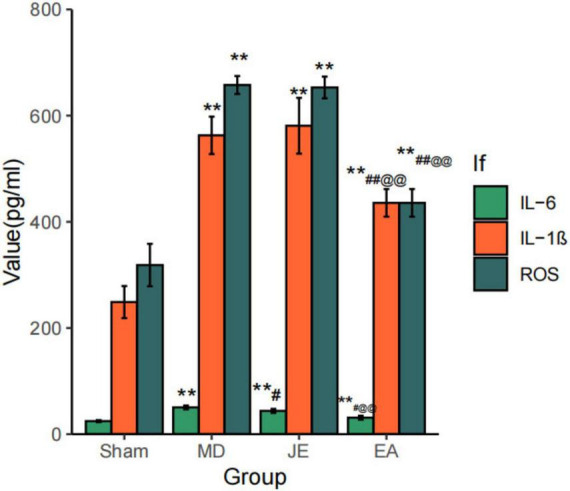
Histograms showing that IL-6, IL-1β and ROS at 4 weeks postoperatively. The Sham group represents the sham surgery control group, the MD group represents the tibial fracture and propofol anesthsia model group, the JE group is the MD treated with sham electric-acupuncture, and the EA group is the MD pretreated with the electric-acupuncture (EA). The IL-6, IL-1β and ROS measures their inflammation in serum. A reduction in IL-6, IL-1β and ROS, where the better of their recovery. (Compare with Sham group, **P < 0.01; compare with MD group, ^#^*P* < 0.05, ^##^*P* < 0.01; compare with JE group, ^@@^*P* < 0.01 *N* = 10/group). The experiment was repeated three times, and data were analyzed using repeated measures ANOVA.

As shown in Figure 8, compared to Sham, MD and JE shows significant higher IL-6, IL-1β and ROS at 4 weeks postoperatively, indicating notable inflammation activity after tibial fracture surgery and propofol anesthsia (*P* < 0.01). However, with electroacupuncture preconditioning, the EA group showed low IL-6 compared with MD and JE (*P* < 0.05), and low IL-1βand ROS (*P* < 0.01), suggesting that electroacupuncture preconditioning could alleviate inflammation after tibial fracture surgery and propofol anesthsia (*P* < 0.01). The raw statistical results are available in Supplementary File 4.

### 3.5 Comparison of intestinal microbiota in each group of rats

#### 3.5.1 Alpha diversity analysis

##### 3.5.1.1 Rarefaction curve

**FIGURE 9:**
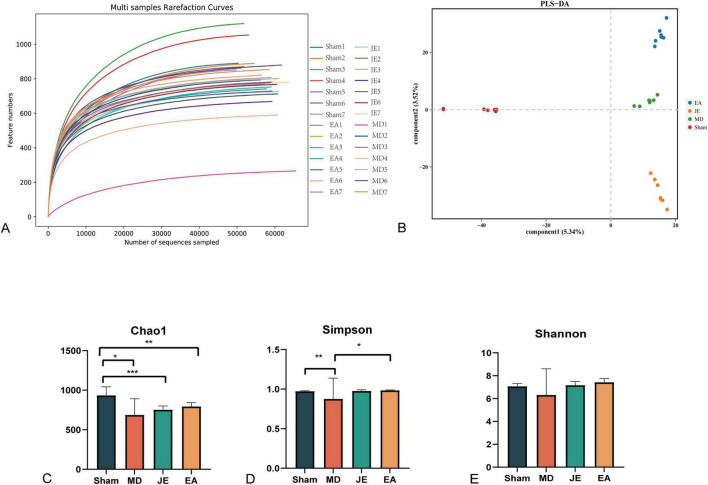
Box plots showing species diversity analysis in each group: **(A)** species dilution curve, **(B)** principal component analysis, **(C)** Chao1 index, **(D)** Simpson index, **(E)** Shannon index, (statistical significance: *P < 0.05, **P < 0.01, ***P < 0.001, *N* = 7/group). The experiment was repeated three times, and data were analyzed using ANOVA.

The rarefaction curve can be used to compare the species richness of samples with different sequencing depths and to assess whether the sample size is adequate. In this study, the rarefaction curve approached a plateau, indicating that the sequencing depth was sufficient and met the data analysis requirements see Figure 9A.

##### 3.5.1.2 Alpha diversity indices

Alpha diversity reflects the richness and diversity of species in a single sample, with several metrics for measurement: Chao1, Shannon, and Simpson. The Chao1 index measures species richness, i.e., the number of species. The Simpson indices are used to measure species diversity. As shown in Figure 9D, compared to the Sham and EA group, Simpson index in MD was reduced (*P* < 0.01, *P* < 0.05, respectively). As shown in Figure 9C, the Chao1 index was significantly reduced in the MD, JE, and EA groups compared to the Sham group (*P* < 0.01 and *P* < 0.05, respectively). As shown in Figure 9E, the Shannon index demonstrated no significant differences among the experimental groups.

##### 3.5.1.3 PLS-DA analysis

PLS-DA (Partial Least Squares Discriminant Analysis) effectively distinguishes between inter-group observations and identifies variables that contribute to the observed group differences. If the samples are separated along the *X*-axis, it suggests that the factor represented by the *X*-axis is the main contributor to the separation. If the samples are also separable along the *Y*-axis, it indicates that the factor represented by the *y*-axis plays a significant role in the separation. As shown in Figure 9B, the horizontal axis represents the principal component scores in the component analysis process (Component 1), which allows for the visualization of differences between groups. The vertical axis represents the orthogonal component scores (Component 2, 3.52%), reflecting the variations within groups (i.e., differences among intra-group samples). In Figure 9B, the Sham group (red), MD group (green), JE group (orange), and EA group (blue) are displayed. The horizontal axis indicates that the gut microbiota composition of the Sham group differs significantly from that of the MD, JE, and EA groups after modeling. The vertical axis shows that the intra-group variation is smallest in the Sham (red) and MD groups, while the JE group exhibits the greatest intra-group variation. This suggests that the modeling intervention significantly affects gut microbiota composition.

#### 3.5.2 Intestinal microbiota structure analysis

**FIGURE 10:**
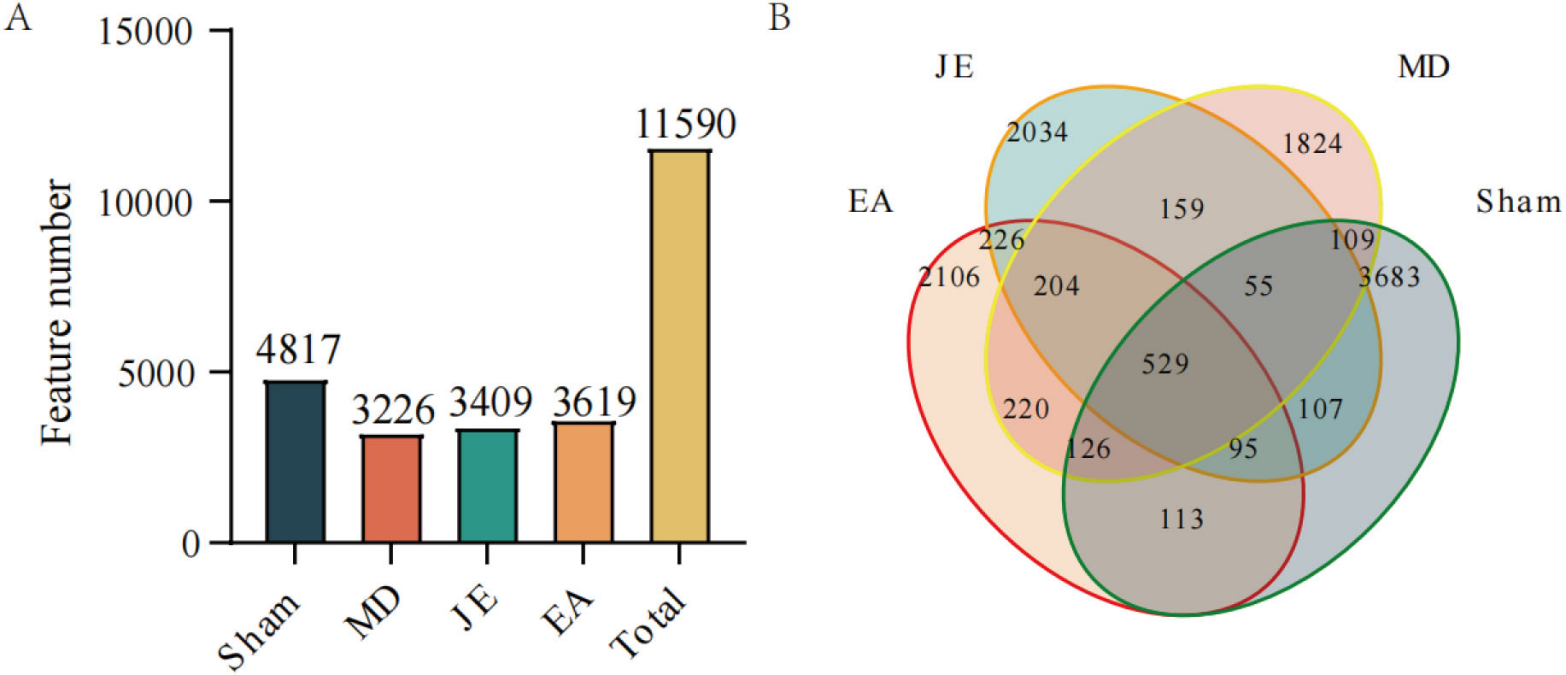
Histograms of OTU distribution in each group. **(A)** OTU number in each group, **(B)** Venn shows same and different OTU (*N* = 7).

As show in Figure 10, Sequences with higher than 97% similarity were grouped into one OTU cluster, Sham group had a total of 4,817 Operational Taxonomic Units (OTUs), OTUs were defined by taxonomy. The MD group had 3,226 OTUs, the JE group had 3,409 OTUs, and the EA group had 3,619 OTUs. Among the four experimental groups, 529 OTUs were common, while the EA group had 2,106 unique OTU.

#### 3.5.3 Phylum-level analysis of intestinal microbiota

**FIGURE 11:**
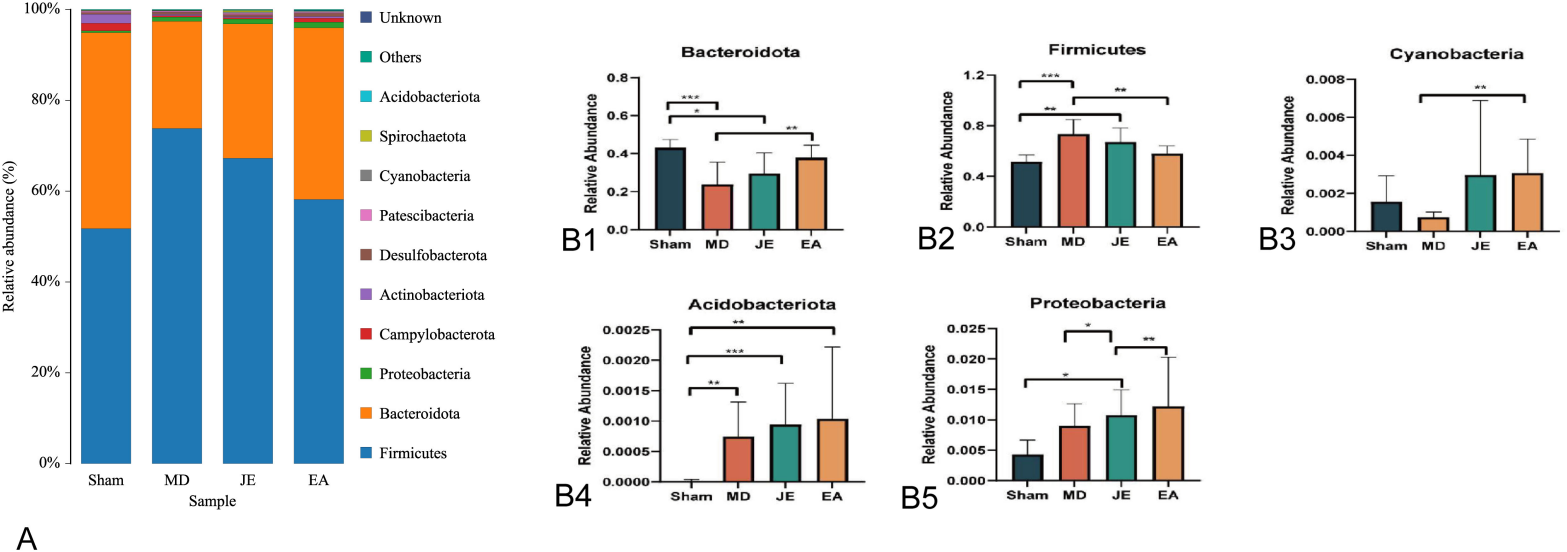
**(A)** Histograms of the relative abundance of colon flora at phylum level. **(B)** Histograms of the relative abundance of the relative abundance of the microbiota of the colon flora at phylum level. The experiment was repeated three times, and data were analyzed using ANOVA. (Statistical significance: *P < 0.05, **P < 0.01, ***P < 0.001; *N* = 7).

As shown in Figure 11, Compared to the Sham group, the relative abundance of Bacteroidota (Bacteroidetes) was reduced in the MD and JE groups (*P* < 0.05), while the relative abundance of Firmicutes and Actinobacteriota was increased (*P* < 0.05). Compared to the MD group, the relative abundance of Bacteroidota and Cyanobacteria increased in the EA group (*P* < 0.05), while the relative abundance of Actinobacteriota was decreased (*P* < 0.05). Compared to the JE group, the relative abundance of Proteobacteria was higher in the EA group (*P* < 0.05).

#### 3.5.4 Genus-level analysis of intestinal microbiota

**FIGURE 12:**
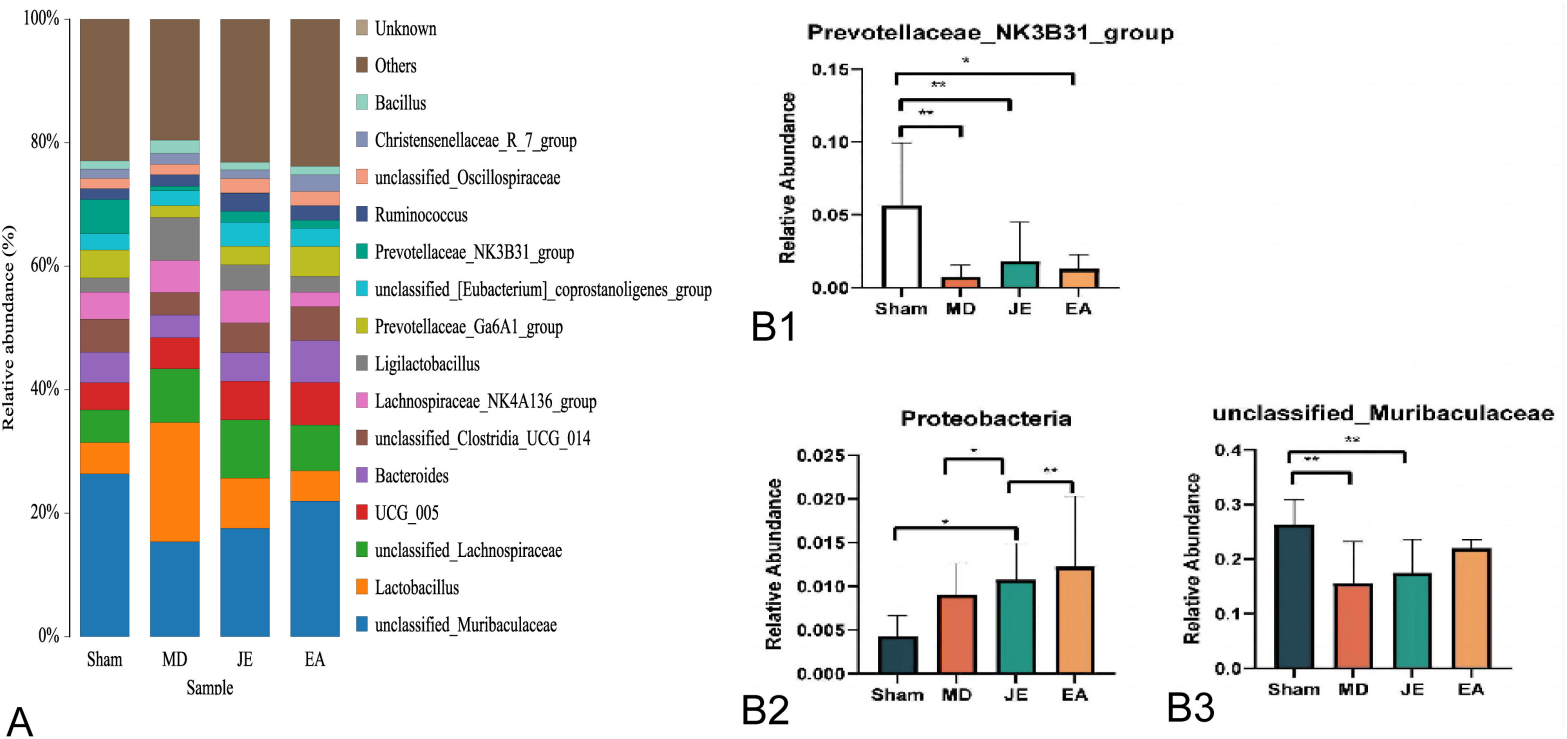
**(A)** Histograms of the relative abundance of colon flora at Genus level. **(B)** Histograms of the relative abundance of the relative abundance of the microbiota of the colon flora at Genus level. The experiment was repeated three times, and data were analyzed using ANOVA. (Statistical significance: *P < 0.05, **P < 0.01; *N* = 7).

As shown in Figures 12A, B, further analysis of the rat intestinal microbiota at the genus level revealed the following results: Compared to the Sham group, the relative abundance of Prvotellaceae_NK3B31 was significantly lower in the MD and JE groups (*P* < 0.01) and decreased in the EA group (*P* < 0.05). The relative abundance of unclassified Muribaculaceae was significantly lower in the MD and JE groups compared to the Sham group (*P* < 0.01).

## 4 Discussion

The present study sought to utilize a combination of propofol anesthesia and tibial fixation surgery to simulate real-world anesthesia and surgical scenarios, with the objective of investigating the cognitive alterations in aged rats following anesthesia and surgery. The findings of this study indicated that the administration of propofol anesthesia and tibial fixation surgery resulted in behavioral changes in aged rats. These alterations included an increase in the latency period in the Morris water maze (MWM), a reduction in crossing times, and a significant decrease in total distance in the open field. Collectively, these changes contributed to the development of postoperative cognitive dysfunction (POCD). However, EA preconditioning effectively reduced water maze latency, increased the number of crossing times, and significantly reduced the incidence of POCD following propofol anesthesia and tibial surgery. Moreover, postoperative immobilization negatively impacts muscle strength and shrinks muscle content, unavoidably leading to muscle atrophy. Our study shows that, 1 week after right tibial surgery, the quadriceps muscle significantly atrophied, especially in the MD and JE groups. The findings of this study demonstrate that EA pretreatment effectively reduces the degree of quadriceps atrophy and promotes muscle hyperplasia post-surgery. At 4 weeks post-surgery, the quadriceps circumference in the electroacupuncture group had almost completely recovered, whereas the quadriceps circumference in the MD and JE groups had not yet returned to pre-surgery levels.

Anesthesia, surgery, and stress impact brain through the gut microbiota (Liufu et al., 2020). Dysbiosis of the gut microbiota may promote the activation of microglial cells in the aging brain, leading to neuroinflammation and the onset of POCD (Zhu et al., 2023). Modulating the gut microbiota has been shown to improve cognitive function (Sochocka et al., 2019). Techniques like EA up-regulates beneficial gut microbiota, reduce BBB and intestinal barrier dysfunction, and mitigate hippocampal inflammation in APP/PS1 rats, thus alleviating POCD (Zhong et al., 2022). Our findings suggest that beside effectively alleviating POCD, EA regulates gut microbiota abundance and composition in the aged rats plays a vital role as follows.

First, the EA group had the highest OTU values, and the Shannon index was significantly elevated. In contrast, the Chao1, ACE, Simpson, and Shannon indices in the MD group all decreased. This suggests that EA pretreatment effectively modulates the gut microbiota diversity in rats with POCD. Studies have shown that a decrease in gut microbiota diversity is associated with cognitive function decline (Łuc et al., 2021; Shi et al., 2021). To further elucidate the role of the gut microbiota in the development of POCD and the effect of EA modulation, we analyze the changes in the abundance of gut microbiota at various taxonomic levels following propofol anesthesia and surgery. The results show that the relative abundance of Phylum Bacteroidota was significantly higher in the EA group compared to the MD group. The imbalance of immune-inflammatory responses and dysregulation of the gut-brain axis, along with increased intestinal epithelial permeability, allow bacteria, viruses, and their neuroactive products to invade, triggering central nervous system inflammation and ultimately leading to cognitive impairment (La Rosa et al., 2018). A significant correlation has been observed between cognitive function scores and changes in gut microbiota abundance in Alzheimer’s disease populations (Wang Q. et al., 2024), which show a marked reduction in gut microbiota diversity. Bacteroidota bacteria are crucial for maintaining gut homeostasis.

Secondly, at the phylum level, the relative abundance of Bacteroidota was significantly reduced in the MD and JE groups, while the EA group showed an increased abundance of Bacteroidota. Phylum Bacteroidota bacteria can reduce the expression of inflammatory cytokines, produce acetate and propionate, and enhance the synthesis of short-chain fatty acids, which play an important role in maintaining the integrity of the intestinal mucosal structure and metabolic function (Parada Venegas et al., 2019). Bacteroidota is a common microbial group in the gut, closely related to human health. Recently, the relationship between Bacteroidota and cognition has become a growing area of research. Some studies have found that certain strains within Bacteroidota can metabolize and produce butyrate, which, through the BBB, can influence the growth and function of neural cells (Wang W. et al., 2024). Additionally, by modulating the immune system and neurotransmitters, Bacteroidota altering cerebral function and cognitive states (Ou et al., 2021).

The relative abundance of Proteobacteria increased in the MD, JE, and EA groups, while the Sham group showed low abundance of Proteobacteria. This suggests a potential relationship between Proteobacteria and cognitive impairment following anesthesia and surgery. Additionally, at the genus level, the relative abundance of Unclassified Lactobacillaceae was significantly reduced in the MD and JE groups (*P* < 0.05). EA preconditioning increased the relative abundance of Unclassified Muribaculaceae at the genus level, which contributed to the reduction in POCD occurrence post-surgery. A meta-analysis from the United States also indicated a significant association between increased Proteobacteria and behavioral changes (Shi et al., 2021). These findings hint that Proteobacteria may play a role in cognitive regulation.

General anesthesia, orthopedic surgery, and other stress stimuli are major risk factors for the occurrence of postoperative cognitive dysfunction (POCD) (Choi et al., 2019; Yang et al., 2024). These stress and inflammatory responses from anesthesia and surgery results in gut microbiota dysbiosis via immune responses, metabolic pathways, microbial abundance and composition disorders. The stress-inflammatory-gut-brain axis exerts its effects on the vulnerable aged brain, leading to POCD, which in turn worsens gut microbiota imbalance. Dysbiosis can induce intestinal inflammation, disrupt the gut barrier, and increase intestinal permeability. Metabolites produced by gut microbiota, such as neurotransmitters and short-chain fatty acids (SCFAs), can influence the levels of relevant metabolites in the brain, thereby regulating brain function and cognition, which contributes to a vicious cycle (Cho et al., 2021). Related studies have also confirmed that gut microbiota imbalance can trigger central nervous system inflammation, increasing the risk of cognitive impairment (Ojeda et al., 2021; Wei et al., 2023). Therefore, correcting gut microbiota imbalance has emerged as a potential therapeutic strategy for POCD.

Acupuncture has been demonstrated to improve cognitive function, enhance learning and memory abilities, and alleviate immune-inflammatory responses in the brain (Hao et al., 2022; Yi et al., 2024), and regulate the abundance and composition of the gut microbiota. Among the gut microbiota, key metabolites produced by phyla like Firmicutes, *Streptococcus*, and *Bifidobacterium* are SCFAs, which can affect the blood-brain barrier, stabilize the neuroenvironment, and influence host cognitive function (Mann et al., 2024). Thus, increased inflammation can lead to the onset and progression of Alzheimer’s disease (AD), manifesting as a decline in learning ability and memory impairment (Rost et al., 2022).

## 5 Conclusion

Electroacupuncture (EA) was associated with improved postoperative cognitive function in aged rats following propofol anesthesia and tibial surgery. These improvements were accompanied by changes in gut microbiota composition, including increased relative abundance of Bacteroidota at the phylum level and Unclassified Muribaculaceae at the genus level. While these findings suggest a potential link between EA and modulation of the gut–brain axis, the results demonstrate associations rather than direct causality. Further mechanistic studies are needed to clarify the underlying pathways.

## Data Availability

The original contributions presented in the study are publicly available. This data can be found here: SRA data: PRJNA1118476 (https://www.ncbi.nlm.nih.gov/sra/PRJNA1118476).
