# Supplementary figures and images for: Electroacupuncture pretreatment ameliorates anesthesia and surgery-induced cognitive dysfunction in aged rats: insights from gut microbiota modulation
Source: Front Microbiol. 2025 Dec 1;16:1642337. doi: 10.3389/fmicb.2025.1642337 (PMC12703379; doi:10.3389/fmicb.2025.1642337)

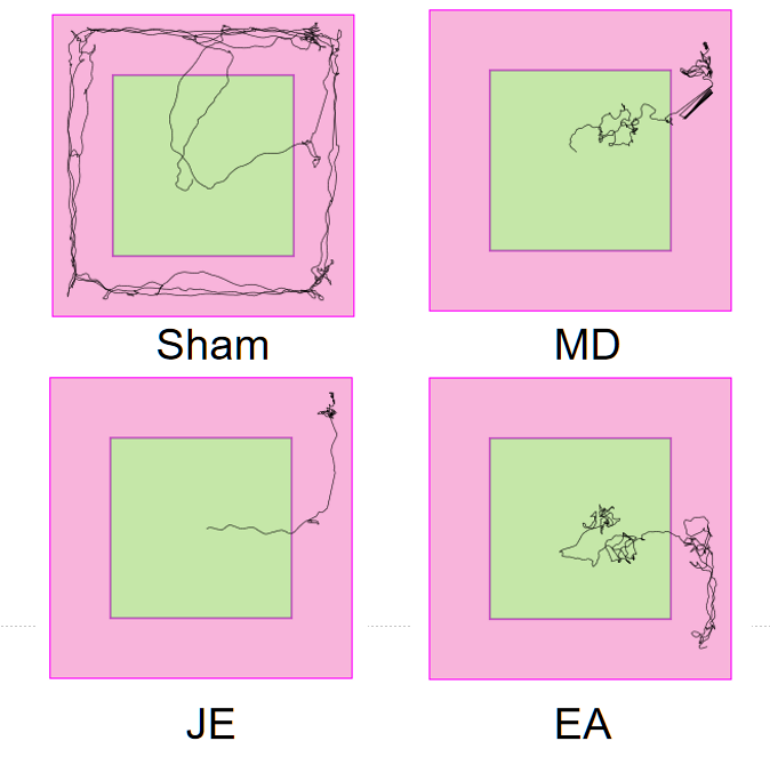

Supplement: Supplementary file 5 [file Image_1.tif]
